# Voluntary running does not reduce neuroinflammation or improve non-cognitive behavior in the 5xFAD mouse model of Alzheimer’s disease

**DOI:** 10.1038/s41598-020-58309-8

**Published:** 2020-01-28

**Authors:** Martina Svensson, Emelie Andersson, Oscar Manouchehrian, Yiyi Yang, Tomas Deierborg

**Affiliations:** Experimental Neuroinflammation Laboratory, Department of Experimental Medical Sciences, Lund University, BMC B11, 22184 Lund, Sweden

**Keywords:** Cognitive neuroscience, Diseases of the nervous system, Neuroimmunology

## Abstract

Physical exercise has been suggested to reduce the risk of developing Alzheimer’s disease (AD) as well as ameliorate the progression of the disease. However, we recently published results from two large epidemiological studies showing no such beneficial effects on the development of AD. In addition, long-term, voluntary running in the 5xFAD mouse model of AD did not affect levels of soluble amyloid beta (Aβ), synaptic proteins or cognitive function. In this follow-up study, we investigate whether running could impact other pathological aspects of the disease, such as insoluble Aβ levels, the neuroinflammatory response and non-cognitive behavioral impairments. We investigated the effects of 24 weeks of voluntary wheel running in female 5xFAD mice (n = 30) starting at 2–3 months of age, before substantial extracellular plaque formation. Running mice developed hindlimb clasping earlier (p = 0.009) compared to sedentary controls. Further, running exacerbated the exploratory behavior in Elevated plus maze (p = 0.001) and anxiety in Open field (p = 0.024) tests. Additionally, microglia, cytokines and insoluble Aβ levels were not affected. Taken together, our findings suggest that voluntary wheel running is not a beneficial intervention to halt disease progression in 5xFAD mice.

## Introduction

Alzheimer’s disease (AD) is the most common form of dementia, affecting around 30 million people worldwide (WHO 2016). Even though cognitive dysfunction is a hallmark of AD, a majority of AD patients also suffer from other, non-cognitive symptoms such as depression and anxiety^1,2^. AD is characterized by accumulation of extracellular amyloid-beta (Aβ) plaques and progressive neurodegeneration. Further, the inflammatory response is also altered in the AD brain^3^. Postmortem studies using AD brains have revealed than pro-inflammatory cytokines, such as IL-1β and IL-6, accumulate around Aβ plaques^4,5^. In addition, microglial activation is increased^6^ and correlates with the Aβ deposition^7,8^. Recently, a genome-wide association study revealed that genetics variants related to increased risk of developing AD are specifically enriched in enhancers of myeloid cells^9^. Interestingly, microglia are capable of phagocytosing Aβ aggregates and, thereby, facilitate Aβ clearance^10^. Contrastingly, neuronal Aβ production can induce cytokines in microglia and this can up-regulate the expression and enzymatic activity of β-secretase, thereby enhancing Aβ production^11^. Thus, it is likely that the microglial response in the AD brain contribute with both protective and harmful effects. Hence, future therapeutic interventions may focus on modulating different aspects of these responses.

Several studies suggest that physical exercise is beneficial by reducing the risk of AD and slowing the progression of the pathology^12–14^. Exercise intervention may improve cognition^15,16^ and ameliorate Aβ levels in patients^17^. Moreover, exercise was associated with larger gray matter volumes in cortex and hippocampus and improved cortical connectivity of cognitive networks in patients with mild cognitive impairment^18,19^. However, many studies show no beneficial effects of exercise on AD^20–23^. We recently investigated how physical activity affects the risk of developing AD in two large study populations (>410 000 participants in total) over an extended period (>20 years) under different conditions^24^. Physical activity did not significantly affect the risk of developing AD in any of our study populations. Hence, we questioned the effect of physical exercise on AD incidence and disease progression.

Several transgenic mouse strains have been developed to model different aspects of AD^25^. The 5xFAD strain is a mouse model with a fast development of AD pathology, showing accumulation of extracellular Aβ plaques and signs of neuroinflammation as early as 2–3 month of age^25–28^. Studies investigating the effects of exercise in other AD models have shown inconsistent results^14^, for example with regard to the effects on Aβ levels^14,29,30^. We have recently shown that 6 months of voluntary running in 5xFAD mice did not result in any beneficial effects on soluble Aβ-levels, synaptic protein levels or cognitive behavior^24^. Interestingly, prior studies in other AD models suggest that exercise may reduce neuroinflammation by reducing microglial activation and levels of pro-inflammatory cytokines^31,32^. Because of its features, we view the 5xFAD model as suitable for studying the effects of exercise on neuroinflammatory and non-cognitive behavioral features of AD. We recently reported on the appearance of neuroinflammation in this model before extracellular amyloid deposition^28^ and the important role of pro-inflammatory microglial galectin-3 in development of pathology and behavioral deficits^33^. In light of the pathological importance of myeloid cells in AD, the aim of this study was to further investigate the effects of 6 months of voluntary wheel running on neuroinflammation and non-cognitive behavior in the 5xFAD model.

## Results

### Voluntary wheel running does not induce a corticosterone stress response

Body weights did not differ between groups at the beginning or end of the study (Supplementary Table 1). Since we previously reported that forced running induces a harmful corticosterone stress response in mice^34^, we controlled for stress induction by the voluntary running intervention used in this study. The fecal corticosterone levels did not significantly differ between sedentary and running mice at baseline or after 19 weeks of exercise intervention (Supplementary Table 2). Both groups displayed decreased levels of corticosterone at the end of the study compared to the baseline levels (Supplementary Table 2, median (IQR) concentrations were 2617 (1699–4455) and 1523 (1331–2205) pg/ml for the sedentary group, Wilcoxon test p = 0.001 and 2167 (1644–4053) and 1506 (1237–1722) pg/ml for the running group, Wilcoxon test p = 0.02).

### Voluntary wheel running affects exploratory and anxious behavior

**Figure 1:**
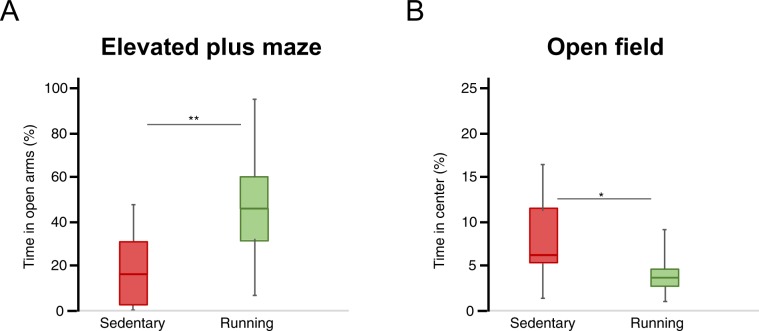
Exploratory behavior in Elevated Plus Maze and Open Field tests. Exploratory behavior is presented as the percentage of time spent in the open arms of the Elevated Plus Maze (**A**) or in the center zone in the Open Field (**B**) tests conducted during weeks 20–22. Box plots represent the median values for each group with interquartile ranges and error bars indicating the minimum and maximum. **Represents p < 0.01 and *represents p < 0.05 (Mann Whitney U-test). For sedentary mice n = 14 and for running mice n = 14.

In the Elevated plus maze, running mice spent significantly more time exploring the open arms compared to their sedentary counterparts (Fig. 1A, median (IQR) 15.2 (3.1–30.9) % and 46.1 (29.2–60.7) % of time respectively, Mann-Whitney U-test p = 0.001). In the open field, running mice spent significantly less time exploring the center compared to sedentary controls (Fig. 1B, median (IQR) 6.3 (5.3–13.0) % and 3.2 (2.3–5.0) % of time, Mann-Whitney U-test p = 0.024). General motor function did not differ between groups as they traveled the same distance both in the Elevated plus maze and Open field (Supplementary Table 3). There was no significant difference in sucrose preference between sedentary and running mice (Supplementary Table 4).

### Voluntary wheel running does not improve motor learning

**Figure 2:**
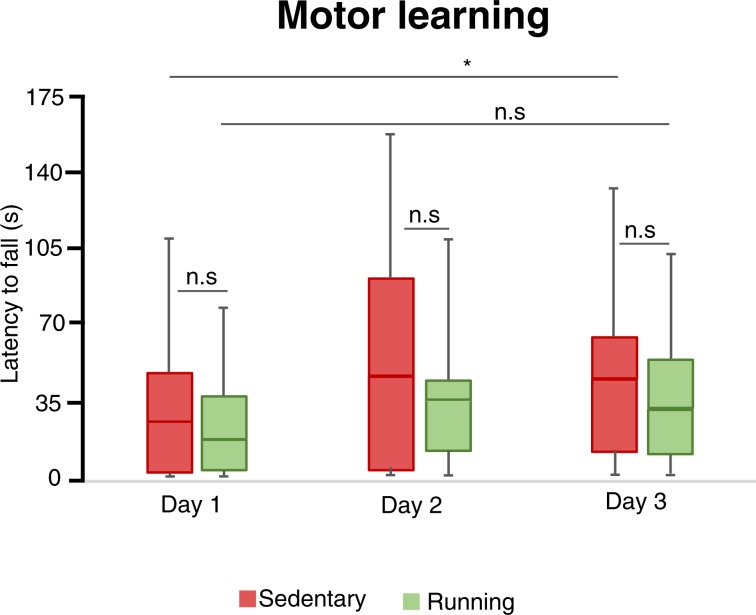
Motor learning in Rotarod test. The latency to fall off the rotarod at different days of training. Box plots represent the median values for each group with interquartile ranges and error bars indicating the minimum and maximum. *Rrepresents p < 0.05 (Wilcoxon test). For sedentary mice n = 14 and for running mice n = 14.

The sedentary mice significantly improved their rotarod performance over time (Fig. 2, median (IQR) 27.8 (1.7–48.0) seconds and 44.3 (12.0–65.0) seconds on day 1 and 3 respectively Friedman test, p = 0.008). In contrast, running mice did not significantly improve over the same amount of time (Fig. 2, median (IQR) 17.2 (4.3–39.3) seconds and 31.5 (13.5–56.5) seconds on day 1 and 3 respectively Friedman test, p = 0.47). However, running mice did not spend significantly less time on the rotarod compared to sedentary littermates on any of the three test occasions. Taken together, these results suggest that voluntary wheel running does not improve motor learning in 5xFAD mice.

### Exercised mice developed hindleg clasping earlier

**Figure 3:**
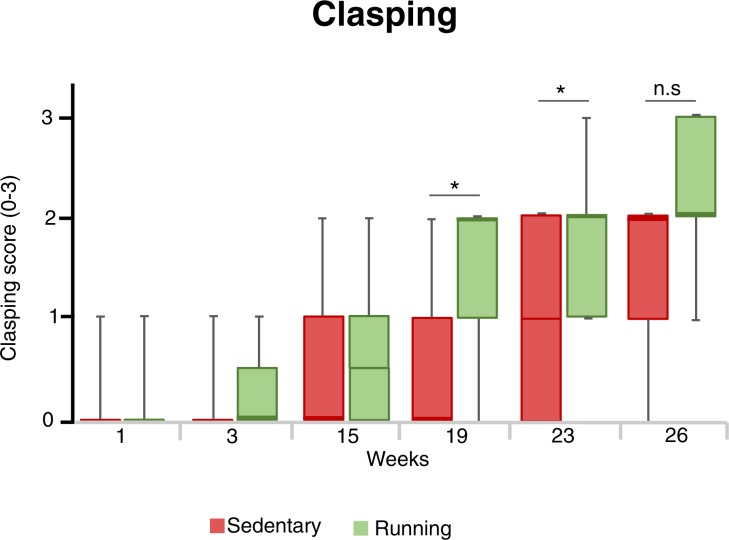
Hindlimb clasping at different time points. The hindlimb clasping scores at different time points. Box plots represent the median values for each group with interquartile ranges and error bars indicating the minimum and maximum. *Represents p < 0.05 (Mann Whitney U-test used for each given time-point). For sedentary mice n = 14, and for running mice n = 14.

To measure the development of sensorimotor dysfunctions in the 5xFAD model, we performed hindlimb clasping tests during experimental weeks 1,3,15,19, 23 and 26 (Fig. 3). There were significant changes in clasping scores in both groups from the beginning to the end of the study (Friedman tests, p<0.001 for both sedentary and running groups). Up to week 15, there was no significant difference in hindleg clasping between sedentary and running mice (week 15, median clasping scores (IQR) were 1 (0–1) and 0.5 (0–1) respectively, Mann-Whitney U-test, p = 0.64). Thereafter, running mice developed hindlimb clasping earlier than sedentary controls (week 19, median clasping scores (IQR)were 1 (0–1) and 2 (1–2) for sedentary and running mice, respectively, Mann-Whitney U-test, p = 0.009. Week 23, median clasping scores (IQR) were 1 (0–2) and 2 (1–2) for sedentary and running mice, respectively, Mann-Whitney U-test, p = 0.029). Nonetheless, at the end of the study, hindlimb clasping scores did not differ significantly between the groups (week 26, median clasping scores (IQR) were 2 (1–2) and 2 (2–3) for sedentary and running mice, respectively, Mann-Whitney U-test, p = 0.20).

### Voluntary wheel running does not ameliorate levels of insoluble Aβ

**Figure 4:**
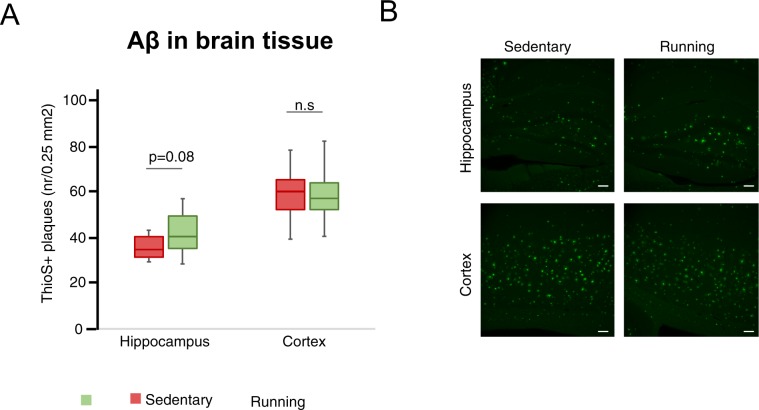
Aβ plaques in hippocampus and cortex. Thioflavin-S-positive Aβ plaques in hippocampus and cortex. Box plots represent the median values for each group with interquartile ranges and error bars indicating the minimum and maximum (**A**). Representative images at 10x, with scale bar representing 100 μm (**B**). p-value from Mann Whitney U-test. For sedentary mice n = 14, and for running mice n = 14.

The levels of different insoluble Aβ species in hippocampus and soluble Aβ species in CSF did not differ between the running and sedentary mice groups (Supplementary Table 5). Further, the number of ThioflavinS-positive amyloid plaques in hippocampus and cortex did not differ significantly between groups (Fig. 4, median plaque numbers (IQR) in hippocampus were 35.2 (29.3–39.7) and 40 (35.7–49.7) for sedentary and running groups respectively, Mann-Whitney U-test, p = 0.077. Median plaque numbers (IQR) in cortex were 60.7 (52.3–65.3) and 55.8 (52.0–63.0) for sedentary and running groups respectively, Mann-Whitney U-test, p = 0.54).

### Voluntary wheel running does not significantly reduce neuroinflammation

**Figure 5:**
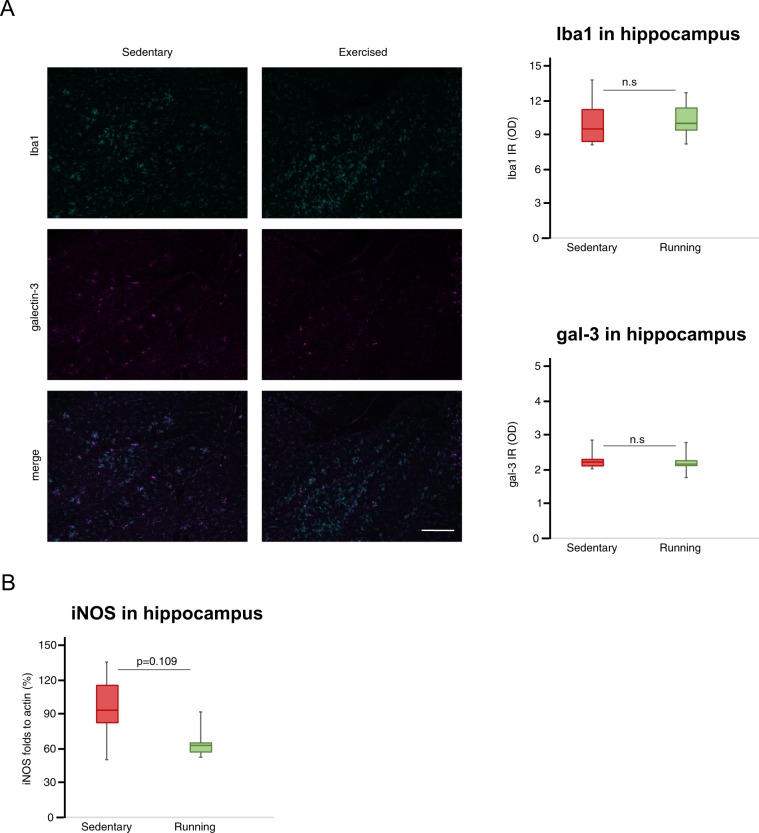
Neuroinflammation in hippocampus. Representative images of the Iba1 (labeling all microglia) and gal-3 (labeling activated microglia) staining in hippocampus at 10x with scale bar representing 200 μm (**A**) and box plots representing the median values of Iba1 and gal-3 intensities (n = 10 + 10). The level of iNOS (n = 6 + 6) in hippocampus normalized to actin (**B**). Box plots represent the median values for each group with interquartile ranges and error bars indicating the minimum and maximum. p-value from Mann Whitney U-test. For sedentary mice n = 6, and for running mice n = 6.

The total amount of microglia in hippocampus was measured by Iba1 immunohistochemistry. Intensity levels of Iba1 did not differ between running and sedentary mice (Fig. 5A). Further, the levels of galectin-3 was not affected by running, as measured using Western blot (Supplementary Table 6) and immunohistochemistry (Fig. 5A). There were no differences in cytokine levels between the groups for any of the cytokines analyzed in serum or hippocampus (Supplementary Table 7). Likewise, the protein levels of NLRP3 (Supplementary Table 6) as well as the levels of iNOS (Fig. 5B, median (IQR) were 94.9 (82.3–116.1) % and 63.2 (57.2–65.4) % for sedentary and running groups, respectively, Mann-Whitney U-test, p = 0.109) in hippocampus did not significantly differ between groups.

## Discussion

In the present study, we investigated the effects of voluntary wheel running on the development of neuroinflammation, insoluble Aβ load and non-cognitive behavioral deficits in the 5xFAD mouse model of AD. Our main findings show that 6 months of voluntary wheel running does not ameliorate these pathological events in 5xFAD mice. On the contrary, running may even aggravate the pathology as our running mice showed increased exploratory behavior and developed sensorimotor hindleg clasping earlier. Furthermore, the running intervention did not reduce insoluble Aβ levels, the total amount of microglia, as measured by Iba1 staining intensity, or pro-inflammatory inflammatory cytokine levels.

Running led to increased exploratory behavior in the Elevated plus maze test and increased anxiety in the Open field test. This may reflect the typical phenotypical differences that this AD model displays compared to wild-type mice in these two tests in sedentary control settings^35,36^. At 8 months of age, 5xFAD mice typically develop increased exploratory behavior in the Elevated plus maze, which correlates with the deposition of Aβ in the brain^35–37^. This increased exploratory behavior has been suggested to reflect disinhibitory tendencies, similar to what is seen in AD patients^35^. Thus, the increased exploratory behavior seen in our running mice might be interpreted as an aggravation of the behavioral dysfunction in this model. However, in this study, we had no direct comparison to wildtype mice. Hence, we cannot know if the behavior we observe in our 5xFAD really deviates from wildtype in our settings, even though existing literature strongly indicate this.

Concurrently, under sedentary conditions, 5xFAD mice have been shown to develop reduced exploratory behavior in Open field as the disease develops^36^. Hence, the increased anxious behavior seen in the Open field in our running mice can also be interpreted as an aggravation of the behavioral dysfunction. Still, we do not have any direct comparison with wildtype mice in our study to conclude this. In addition, we have previously shown that anxious behavior in Open Field is associated with increased corticosterone levels in feces collected during this test^34^. Since the corticosterone levels in feces collected during the Open Field test performed at 8 months of age in our study did not differ, it is possible that the readout of this test does not really reflect the anxiety levels during that day. Therefore, we should be careful with conclusions drawn from this test.

Hindlimb clasping and motor deficits normally develops at 9–12 months in the 5xFAD model and are suggested to reflect the Aβ accumulation and damage in spinal cord motor neurons^35,38^. In our study, running mice developed clasping earlier and motor performance and learning in the rotarod was not improved by the running intervention. Therefore, it is tempting to speculate that the increased clasping behavior in the running mice reflects a faster development of the pathology in the central nervous system. However, we do not control for development hindlimb clasping in wildtype mice since there is already robust evidence that wildtype mice do not develop this abnormal clasping behavior. Importantly, the distance traveled in the Open field and Elevated plus maze tests did not differ between the groups, indicating that the motor deficits in the clasping test did not bias the outcome of these anxiety tests.

Although many experimental studies demonstrate reduced Aβ levels in the brain after exercise^39^, we could not detect any statistically significant changes on the levels of soluble Aβ^24^ and insoluble Aβ. Interestingly, we observed a nonsignificant trend towards increased Aβ plaques in the hippocampus (p = 0.08) of running mice, in line with the effects of running found in a model of cerebral amyloid angiopathy^40^. In addition, other studies showed no effects of exercise on Aβ levels in mouse models of AD^41,42^. Further, a patient study with exercise intervention found no effects on Aβ levels in CSF^43^, similar to what we observed in CSF from our 5xFAD mice. Hence, the clinical benefits regarding the effect of exercise on Aβ pathology in AD indicated in other experimental studies can be questioned.

Our group has previously showed that the 5xFAD model displays increased levels of inflammatory cytokines and neuroinflammation as early as 2–3 months of age, the same time period when the first Aβ plaques can be observed^28^. Further, manipulating cytokine and galectin-3 levels has been shown to affect Aβ pathology in the 5xFAD model^33,44^. Since exercise is known to affect the levels of several cytokines^32,42,45^, these studies led us to introduce the running intervention early in our study. However, running did not affect brain or blood cytokine levels or total microglia, as measured by Iba1 staining intensity, in our mice. Further, we could not detect any significant effects on other inflammatory markers, such as galectin-3, iNOS and NLRP3. Even though running tended to reduce iNOS levels, the effect was not statistically significant. The failure of our running intervention to affect the inflammatory reaction in the brains of our mice may be one explanation as to why the intervention did not influence Aβ accumulation or behavioral outcome, although it is interesting to note that exercise ameliorated pathology and cognitive dysfunction in other AD models without affecting cytokine levels^46^.

Taken together, running exercise did not ameliorate any pathological hallmarks in our study. We do not compare with wildtype mice in our study. Still, our results indicate that a running intervention may aggravate the disease phenotype, such as increasing exploratory behavior in the Elevated plus maze, shown to be an abnormal behavior compared to wildtype in other studies. Similarly, our previous publication revealed that the intervention also aggravated cognition in the 5xFAD model^24^. Nevertheless, numerous studies have demonstrated beneficial results of exercise on AD pathology in other mouse models of the disease^14,39^. These differences may be due to several factors. First, the 5xFAD model is an aggressive model with a fast progression and a genetically driven pathology whereas most AD mouse models have a slower progression^25^. Thus, the aggressive pathology in 5xFAD mice might be more difficult to impede compared to the slower development of AD-like pathology in other models. Second, discrepancies between studies may be explained by the duration and timing of the intervention and sample collection. Many studies, compared to this study, investigate the effects of exercise over a shorter time period, making it difficult to draw conclusions about the effects of a long-term, active lifestyle initiated before pathology develops. In our study, the running intervention is started at two months of age, when AD pathology begins to develop in 5xFAD mice. In addition, our mice exercised for six months, until eight months of age, when this model has fully developed the pathology. Moreover, the mice in many exercise intervention studies are socially isolated, which some researchers suggest, may influence the results^47^. Importantly, this was not an issue in our study as our mice were housed in pairs.

Nevertheless, Choi *et al*. recently reported that running was beneficial in this model and reduced Aβ levels and improved cognition^48^. We have previously observed that forced running paradigms may induce stress in mice, which can aggravate the pathology^34^. Therefore, we compared corticosterone levels from running mice with the sedentary controls both before and after the running intervention. We did not find any signs of stress in our running mice as the corticosterone levels did not differ between groups. Interestingly, the corticosterone levels even decreased significantly in both running and sedentary groups at the end of our study. Moreover, our study followed the mice until 8 months of age, whereas the study by Choi *et al*. followed the mice until 6 months of age. Hence, it is possible that exercise may have beneficial effects in this model when measured at an earlier timepoint but cannot counteract the pathology at more advanced stages. Additionally, Choi *et al*. do not investigate effects on neuroinflammation or anxiety in their study, so it is impossible to know how these aspects were affected. Unlike their beneficial effects, we continuously monitored hindlimb clasping in our study and observed that running accelerated the development of this pathological behavior. The reasons for the discrepancies between our study and the study presented by Choi *et al*. are not likely to be explained by the genetic background as they use the same background strain as the 5xFAD mice used in our study. Discrepancies between our studies are more likely to be attributable to differences in the running protocol. Our running mice had *ad libitum* access to running wheels in their home cage, whereas the mice in Choi *et al*. study were only allowed 3 hours of running per day when they were transferred to another cage for their exercise intervention. In addition, while we house our mice in pairs, their mice also seem to be singly housed, which may induce depression and, in turn, affect behavior of mice^49^. Thus, it is possible that running counteracts some of the negative effects caused by single-housing in that study.

To the best of our knowledge, the ability of exercise to aggravate AD pathology has not been reported before. Rather, a handful of studies, using other AD models, show no effects of exercise on cognition^30^. This may be due to publication bias since it is less likely for a study reporting primarily negative data to be accepted in respected scientific journals.

In addition to the above-mentioned limitations, our study includes other obstacles regarding the translation of our results to the clinic. First, animal models do not fully recapitulate all hallmarks of AD. Second, the 5xFAD model has a genetically driven, aggressive form of the pathology, whereas the majority of human AD cases are sporadic^50,51^. Hence, we cannot exclude the possibility of exercise to be protective for development of the sporadic forms of the disease in AD mice with a slower progression, modelling most of the cases seen in the clinic.

## Conclusions

Our study shows that running exercise may not only lack protective effects on the development of the AD phenotype in 5xFAD mice but may also accelerate and aggravate it.

## Methods

### Animals

Animal experiments were approved by the Malmö/Lund animal ethics committee (2012, Dnr: M427-12) and performed in accordance with the Directive of the European Parliament. The setup of this study has been described before^24^. As single housing can affect behavior^49^, we housed our mice in pairs. Since housing male mice together may induce aggressive behavior influencing the outcome as described previously^34^, we only used females.

**Figure 6:**
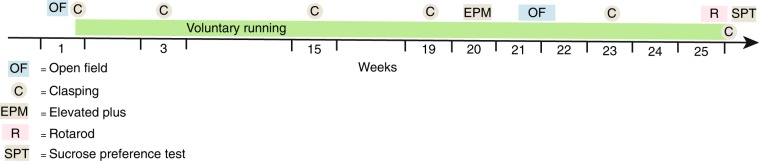
Experimental design. Mice had access to running wheels during experimental weeks 2–25. Before the introduction of exercise intervention, during week 1, Open Field test (OF) and Clasping test (C) were conducted. Clasping tests were then repeated in weeks 3, 15, 19, 23 and 26. During week 20 Elevated Plus maze tests (EPM) were conducted. During weeks 21–22, Open Filed tests were performed. During weeks 25–26 Rotarod tests (R) were conducted. During the last week, week 26, a sucrose Preference test (SPT) was performed before the mice were sacrificed (+) to collect brain, blood and CSF samples.

Briefly, we used 30 female 5xFAD mice on a C57Bl/6*SJL background, obtained from Jackson laboratories, aged 9–12 weeks at the beginning of the study. Mice were housed in pairs, and each pair was randomly assigned to one of two groups: mice with access to a running wheel (“running mice”) or mice without access to a running wheel (“sedentary mice”). There was no significant difference in body weights between groups at the beginning and end of the study (Supplementary Table 1). The experimental outline can be seen in Fig. 6.

### Voluntary wheel running exercise

At 9–12 weeks of age, mice were caged with (n = 16) or without (n = 14) running wheels for 24 weeks, until the end of the study. Running mice had *ad libitum* access to low-profile wireless running wheels (med-associates) in their home cage. The running distance was measured telemetrically to control that mice were running (Supplementary Fig. S1). Visual observation during the active period confirmed that running mice were significantly more active than sedentary mice in their home cages.

### Behavioral tests

#### Open field test

In order to evaluate the locomotion and anxiety levels of the mice, the Open field test was conducted as described previously^34^. The test was performed one week prior to introducing the running wheels as well as after 19 weeks of voluntary wheel running. The mice were placed in an empty white box (45 × 45 cm) and allowed to freely explore it for 10 minutes. An automated behavioral system (SMART, Panlab, Barcelona, Spain) was used to measure the velocity of the movements, the distance traveled and the time spent in the center and periphery of the box. More time spent away from the center zone was regarded as a sign of anxiety. The box was cleaned with ethanol followed by water before each mouse was introduced to the Open field arena.

#### Clasping scoring

Throughout the study, hindlimb clasping behavior, a pathological motor reflex, was assessed regularly at six different time points (experimental weeks 1, 3, 15, 19, 23 and 26). The mice were held near the base of their tail and allowed to hang free for 30 seconds, during which the clasping behavior was recorded and scored. Clasping was scored using a scale between 0 and 3, where 0 represented no clasping (normal), 1 represented initial signs of clasping or only clasping of one hindleg for at least 50% of the time, 2 represented clasping of both hindlegs for at least 50% of the time, and 3 represented clasping of both hindlegs for nearly 100% of the time as described previously^52^.

#### Elevated plus maze test

To examine exploratory and anxiety-like behavior, the mice were subjected to elevated plus maze test after 18 weeks of running. The elevated plus maze apparatus consisted of two open arms and two closed arms (29 × 6 cm). The entire maze was elevated about 40 cm from the floor. Each mouse was placed in the center of the maze with their head facing towards the open arm. During a 5-min test, the time spent in the open arms and the total distance traveled were recorded from above using the SMART system. A healthy mouse is curious and spend more time exploring the open arms, while a mouse with anxiety spends most of its time in the closed arms^53,54^.

#### Rotarod test

To examine motor coordination and balance, mice were subjected to the rotarod test after 23 weeks of running. The rotarod apparatus (8200 model, Letica Scientific Instruments, LE, US) consists of a rotating spindle (3 cm diameter, 15 cm long base) with five individual, 3 cm-wide, compartments allowing for up to five mice to be tested simultaneously. Mice were placed on the rotating rod and tested by increasing the rotating speed from 4 to 40 rpm over 300 seconds. The mean time that a mouse remained on the rotarod was recorded and calculated from three trials. The mice were allowed to rest in their home cage for at least 45 min between trials. The mice were subjected to the rotarod test for three days in order to examine their motor learning abilities.

#### Sucrose preference test

The Sucrose preference test is described in Supplementary Methods.

### Fecal corticosterone levels

Corticosterone measurements are described in Supplementary Methods.

### Collection of samples

After 24 weeks of voluntary wheel running, mice were sacrificed to collect samples. The mice were anesthetized with isoflurane and CSF was collected from cisterna magna using a transparent glass capillary checking for no contamination of blood when mice were under deep anesthesia. CSF samples were snap-frozen immediately in dry ice and stored at −80 °C until analysis. Afterwards, the mice were euthanized and blood samples were collected through cardiac puncture. Blood samples were kept at room temperature for 25 min and then stored on ice for a few hours until the samples were centrifuged at 1300 g at 4 °C for 10 min. The serum supernatants were collected and stored at −80 °C until analysis. Mice were perfused with saline solution before the brains were removed. The right hemisphere was fixed in 4% paraformaldehyde in phosphate buffer for 24 hours before being stored in 30% sucrose solution at 4 °C until analysis. From the left hemisphere, the cerebellum, hippocampus and cortex were dissected, snap-frozen in dry ice and stored at −80 °C until analysis.

### Immunohistochemistry

Sagittal brain sections (30 μm) were prepared from the right hemisphere as previously described^24^.

#### Aβ plaques in cortex and hippocampus

Amyloid plaques were labeled with 0.5% Thioflavin S. Briefly, Thioflavin S was dissolved in ddH_2_O and filtered through a 0.22 μm syringe filter. Sections were incubated during 5 min, rinsed for 3*10 min in PBS and mounted in aqueous mounting media. Three sections per brain (lateral 0.84–1.2 mm) were analyzed using an epifluorescence (Nikon Eclipse 80i microscope, Europe) microscope. The thioflavinS-positive plaques were counted in a 0.25 mm^2^ area within regions of interest; dentate gyrus/CA4 in hippocampus and cortical layer 4 and 5 in the neocortex area above the lateral ventricle.

#### Microglia in hippocampus

Microglia were labeled with primary antibodies against Iba1 (rabbit, Wako, product nr 27981192, 1:750) and galectin-3 (goat, R&D, product nr AF1197, 1:1000) and secondary Alexa Fluor antibodies against rabbit (647 nm, Invitrogen, product nr A32795, 1:500) and goat (488 nm, Invitrogen, product nr A-11055, 1:500). Three sections per brain (lateral 0.84–1.2 mm) were imaged using an epifluorescence microscope (Nikon Eclipse 80i microscope, Europe). The immunofluorescence intensity was analyzed using ImageJ from 10x pictures of the dentate gyrus/CA4 in hippocampus.

### Homogenization of brain tissue

The hippocampus was homogenized to extract proteins in three different fractions. The first fraction containing soluble proteins was extracted by grinding the tissue 20 times with a dounce homogenizer in 120 μl of TBS buffer (20 mM Tris-HCl, 137 mM NaCl, pH 7.6) containing protease and phosphatase inhibitors. The homogenate was incubated 30 min on ice before it was centrifuged at 14 000 g at 4 °C for 30 min after which the supernatant was collected. To obtain the second fraction containing the membrane-bound proteins, the remaining pellet was re-suspended in 120 μl of TBS with protease and phosphatase inhibitors and 1% Triton-X100. The suspension was incubated for 30 min on ice before it was centrifuged at 14 000 g at 4 °C for 30 min and the supernatant was collected. The third fraction containing insoluble protein aggregates, such as Aβ plaques, was obtained by re-suspending the remaining pellet in 120 μl of 70% formic acid. The suspension was then sonicated at an amplitude of 60% with repeating 10-second pulses followed by 10-second pause for a total of 2 minutes before it was centrifuged at 14 000 g at 4 °C for 30 min. The supernatant was neutralized 1:20 in 1 M Tris. Protein concentrations were determined (Pierce microplate BCA Protein Assay kit for the first and second fraction and the Pierce Coommassie Plus Assay kit for the third fraction). Samples were stored at −80**°**C until use.

### Multiplex ELISA

Cytokine and Aβ ELISA are described in Supplementary Methods.

### Western blotting

Protein levels of iNOS, galectin-3 and NLRP3 in the second fraction of homogenized hippocampus were measured by Western blot. Briefly, samples were loaded into 4–20% Mini-Protean TGX precast pels (Bio-Rad), then transferred to nitrocellulose membranes (Bio-Rad) using the Trans-Blot Turbo System (Bio-Rad). The membranes were then blocked with 3% casein (Sigma-Aldrich) diluted in PBS. After blocking, the membranes were incubated with primary antibodies against galectin-3 (1:3000, AF1197, R&D Systems), iNOS (1:500, SC650, Santa Cruz) and NLRP3 (1:1000, AG-20B-0014-C100, Adipogen) at 4 **°**C over night. The membranes were then incubated with peroxidase-conjugated secondary antibodies (1:5000, Vector Labs) and the blots were developed using Clarity Western ECL Substrate (Bio-Rad). Protein levels were normalized to beta-actin (1:10000, A3854, Sigma).

### Statistical analyses

All statistical analyses were performed using SPSS version 22.0. Body weight and cytokine data was considered normally distributed and analyzed with student’s T-tests. Data obtained from brain tissue stains and western blots were analyzed with Mann-Whitney U-tests. To compare the behavioral performance data between the sedentary and running groups, Mann-Whitney U-tests were used. To compare evolution of Rotarod and Clasping behavior over time within groups Friedman tests were used. For specific time-points of these tests, groups were compared with Mann-Whitney U-tests. To compare pre- and post-intervention of corticosterone levels Wilcoxon tests were used. P-values below 0.05 were considered statistically significant.

## Supplementary information

Supporting Information.

## References

[1] Tokuchi R (2016). Differences between the behavioral and psychological symptoms of Alzheimer’s disease and Parkinson’s disease. J Neurol Sci.

[2] Assal F, Cummings JL (2002). Neuropsychiatric symptoms in the dementias. Curr Opin Neurol.

[3] Calsolaro V, Edison P (2016). Neuroinflammation in Alzheimer’s disease: Current evidence and future directions. Alzheimers Dement.

[4] Griffin WS (1989). Brain interleukin 1 and S-100 immunoreactivity are elevated in Down syndrome and Alzheimer disease. Proc Natl Acad Sci USA.

[5] Hull M, Berger M, Volk B, Bauer J (1996). Occurrence of interleukin-6 in cortical plaques of Alzheimer’s disease patients may precede transformation of diffuse into neuritic plaques. Ann NY Acad Sci.

[6] Edison P (2008). Microglia, amyloid, and cognition in Alzheimer’s disease: An [11C](R)PK11195-PET and [11C]PIB-PET study. Neurobiol Dis.

[7] Fan Z, Okello AA, Brooks DJ, Edison P (2015). Longitudinal influence of microglial activation and amyloid on neuronal function in Alzheimer’s disease. Brain.

[8] Fan Z (2015). Influence of microglial activation on neuronal function in Alzheimer’s and Parkinson’s disease dementia. Alzheimers Dement.

[9] Novikova, G. *et al*. Integration of Alzheimer’s disease genetics and myeloid genomics reveals novel disease risk mechanisms. *BioRxiv*, 10.1101/694281 (2019).

[10] Terwel D (2011). Critical role of astroglial apolipoprotein E and liver X receptor-alpha expression for microglial Abeta phagocytosis. J Neurosci.

[11] Chen CH (2012). Increased NF-kappaB signalling up-regulates BACE1 expression and its therapeutic potential in Alzheimer’s disease. Int J Neuropsychopharmacol.

[12] Rovio S (2005). Leisure-time physical activity at midlife and the risk of dementia and Alzheimer’s disease. Lancet Neurol.

[13] Hamer M, Chida Y (2009). Physical activity and risk of neurodegenerative disease: a systematic review of prospective evidence. Psychol Med.

[14] Ryan SM, Kelly AM (2016). Exercise as a pro-cognitive, pro-neurogenic and anti-inflammatory intervention in transgenic mouse models of Alzheimer’s disease. Ageing Res Rev.

[15] Yaguez L, Shaw KN, Morris R, Matthews D (2011). The effects on cognitive functions of a movement-based intervention in patients with Alzheimer’s type dementia: a pilot study. Int J Geriatr Psychiatry.

[16] Palleschi L (1996). Effect of aerobic training on the cognitive performance of elderly patients with senile dementia of Alzheimer type. Arch Gerontol Geriatr.

[17] Liang KY (2010). Exercise and Alzheimer’s disease biomarkers in cognitively normal older adults. Ann Neurol.

[18] Köbe Theresa, Witte A.Veronica, Schnelle Ariane, Lesemann Anne, Fabian Sonja, Tesky Valentina A., Pantel Johannes, Flöel Agnes (2016). Combined omega-3 fatty acids, aerobic exercise and cognitive stimulation prevents decline in gray matter volume of the frontal, parietal and cingulate cortex in patients with mild cognitive impairment. NeuroImage.

[19] Ahlskog JE, Geda YE, Graff-Radford NR, Petersen RC (2011). Physical exercise as a preventive or disease-modifying treatment of dementia and brain aging. Mayo. Clin. Proc..

[20] Yamada M (2003). Association between dementia and midlife risk factors: the Radiation Effects Research Foundation Adult Health Study. J Am Geriatr Soc.

[21] Sabia S (2017). Physical activity, cognitive decline, and risk of dementia: 28 year follow-up of Whitehall II cohort study. BMJ.

[22] Najar J (2019). Cognitive and physical activity and dementia: A 44-year longitudinal population study of women. Neurology.

[23] Ravaglia G (2008). Physical activity and dementia risk in the elderly: findings from a prospective Italian study. Neurology.

[24] Hansson O (2019). Midlife physical activity is associated with lower incidence of vascular dementia but not Alzheimer’s disease. Alzheimers Res Ther.

[25] Webster SJ, Bachstetter AD, Nelson PT, Schmitt FA, Van Eldik LJ (2014). Using mice to model Alzheimer’s dementia: an overview of the clinical disease and the preclinical behavioral changes in 10 mouse models. Front Genet.

[26] Landel V (2014). Temporal gene profiling of the 5XFAD transgenic mouse model highlights the importance of microglial activation in Alzheimer’s disease. Mol Neurodegener.

[27] Aytan N (2016). Fingolimod modulates multiple neuroinflammatory markers in a mouse model of Alzheimer’s disease. Sci Rep.

[28] Boza-Serrano A, Yang Y, Paulus A, Deierborg T (2018). Innate immune alterations are elicited in microglial cells before plaque deposition in the Alzheimer’s disease mouse model 5xFAD. Sci Rep.

[29] Moore KM (2016). A spectrum of exercise training reduces soluble Abeta in a dose-dependent manner in a mouse model of Alzheimer’s disease. Neurobiol Dis.

[30] Xu ZQ (2013). Aerobic exercise combined with antioxidative treatment does not counteract moderate- or mid-stage Alzheimer-like pathophysiology of APP/PS1 mice. CNS Neurosci Ther.

[31] Xiong JY (2015). Long-term treadmill exercise improves spatial memory of male APPswe/PS1dE9 mice by regulation of BDNF expression and microglia activation. Biol Sport.

[32] Nichol KE (2008). Exercise alters the immune profile in Tg2576 Alzheimer mice toward a response coincident with improved cognitive performance and decreased amyloid. J Neuroinflammation.

[33] Boza-Serrano A (2019). Galectin-3, a novel endogenous TREM2 ligand, detrimentally regulates inflammatory response in Alzheimer’s disease. Acta Neuropathol.

[34] Svensson M (2016). Forced treadmill exercise can induce stress and increase neuronal damage in a mouse model of global cerebral ischemia. Neurobiol Stress.

[35] Jawhar S, Trawicka A, Jenneckens C, Bayer TA, Wirths O (2012). Motor deficits, neuron loss, and reduced anxiety coinciding with axonal degeneration and intraneuronal Abeta aggregation in the 5XFAD mouse model of Alzheimer’s disease. Neurobiol Aging.

[36] Schneider F, Baldauf K, Wetzel W, Reymann KG (2014). Behavioral and EEG changes in male 5xFAD mice. Physiol Behav.

[37] Peters Owen M., Shelkovnikova Tatyana, Tarasova Tatiana, Springe Signe, Kukharsky Michail S., Smith Gaynor A., Brooks Simon, Kozin Sergey A., Kotelevtsev Yury, Bachurin Sergey O., Ninkina Natalia, Buchman Vladimir L. (2013). Chronic Administration of Dimebon does not Ameliorate Amyloid-β Pathology in 5xFAD Transgenic Mice. Journal of Alzheimer's Disease.

[38] O’Leary TP, Robertson A, Chipman PH, Rafuse VF, Brown RE (2018). Motor function deficits in the 12 month-old female 5xFAD mouse model of Alzheimer’s disease. Behav Brain Res.

[39] Intlekofer KA, Cotman CW (2013). Exercise counteracts declining hippocampal function in aging and Alzheimer’s disease. Neurobiol Dis.

[40] Robison LS (2019). Long-term voluntary wheel running does not alter vascular amyloid burden but reduces neuroinflammation in the Tg-SwDI mouse model of cerebral amyloid angiopathy. J Neuroinflammation.

[41] Garcia-Mesa Y (2011). Physical exercise protects against Alzheimer’s disease in 3xTg-AD mice. J Alzheimers Dis.

[42] Parachikova A, Nichol KE, Cotman CW (2008). Short-term exercise in aged Tg2576 mice alters neuroinflammation and improves cognition. Neurobiol Dis.

[43] Steen Jensen C (2016). Cerebrospinal Fluid Amyloid Beta and Tau Concentrations Are Not Modulated by 16 Weeks of Moderate- to High-Intensity Physical Exercise in Patients with Alzheimer Disease. Dement Geriatr Cogn Disord.

[44] Paouri E, Tzara O, Zenelak S, Georgopoulos S (2017). Genetic Deletion of Tumor Necrosis Factor-alpha Attenuates Amyloid-beta Production and Decreases Amyloid Plaque Formation and Glial Response in the 5XFAD Model of Alzheimer’s Disease. J Alzheimers Dis.

[45] Souza Leandro C., Filho Carlos B., Goes André T. R., Fabbro Lucian Del, de Gomes Marcelo G., Savegnago Lucielli, Oliveira Mauro Schneider, Jesse Cristiano R. (2013). Neuroprotective Effect of Physical Exercise in a Mouse Model of Alzheimer’s Disease Induced by β-Amyloid1–40 Peptide. Neurotoxicity Research.

[46] Belarbi K (2011). Beneficial effects of exercise in a transgenic mouse model of Alzheimer’s disease-like Tau pathology. Neurobiol Dis.

[47] Hatchard T, Ting JJ, Messier C (2014). Translating the impact of exercise on cognition: methodological issues in animal research. Behav Brain Res.

[48] Choi Se Hoon, Bylykbashi Enjana, Chatila Zena K., Lee Star W., Pulli Benjamin, Clemenson Gregory D., Kim Eunhee, Rompala Alexander, Oram Mary K., Asselin Caroline, Aronson Jenna, Zhang Can, Miller Sean J., Lesinski Andrea, Chen John W., Kim Doo Yeon, van Praag Henriette, Spiegelman Bruce M., Gage Fred H., Tanzi Rudolph E. (2018). Combined adult neurogenesis and BDNF mimic exercise effects on cognition in an Alzheimer’s mouse model. Science.

[49] Berry A (2012). Social deprivation stress is a triggering factor for the emergence of anxiety- and depression-like behaviours and leads to reduced brain BDNF levels in C57BL/6J mice. Psychoneuroendocrinology.

[50] Barber RC (2012). The genetics of Alzheimer’s disease. Scientifica (Cairo).

[51] Thies William, Bleiler Laura (2012). 2012 Alzheimer's disease facts and figures Alzheimer's Association ∗. Alzheimer's & Dementia.

[52] Guyenet, S. J. *et al*. A simple composite phenotype scoring system for evaluating mouse models of cerebellar ataxia. *J Vis Exp*, 10.3791/1787 (2010). 10.3791/1787 PMC3121238 20495529

[53] Lister RG (1987). The use of a plus-maze to measure anxiety in the mouse. Psychopharmacology (Berl).

[54] Walf AA, Frye CA (2007). The use of the elevated plus maze as an assay of anxiety-related behavior in rodents. Nat Protoc.

