# Supplementary material for: Voluntary running does not reduce neuroinflammation or improve non-cognitive behavior in the 5xFAD mouse model of Alzheimer’s disease
Source: Sci Rep. 2020 Jan 28;10:1346. doi: 10.1038/s41598-020-58309-8 (PMC6987124; doi:10.1038/s41598-020-58309-8)
Supplement: Supplementary file 1 — Supporting Information. [file 41598_2020_58309_MOESM1_ESM.pdf]

Voluntary running does not reduce neuroinflammation or improve non-cognitive behavior in the 5xFAD mouse model of Alzheimer's disease

Martina Svensson[1*], Emelie Andersson[1], Oscar Manouchehrian[1], Yiyi Yang[1], Tomas Deierborg[1*]

1. Experimental Neuroinflammation Laboratory, Department of Experimental Medical Sciences, Lund University, BMC B11, 22184 Lund, Sweden

Supplementary Data

Supplementary Table 1- Body weights

	**Sedentary** (Mean±SD)	**Running** (Mean±SD)	**T-test (unpaired) Sedentary vs. Exercised**
**Start Weight (g)**	16.8±1.1	16.1±1.3	P=0.14
**Final Weight (g)**	23.4±1.3	22.6±3.0	P=0.35
**Weight gain (%)**	40±11	40±16	P=0.98

Supplementary Table 2- Corticosterone levels in feces (ELISA)

	**Sedentary** Median (IQR)	**Running** Median (IQR)	**Mann Whitney U-test Sedentary vs. Running**
**Baseline levels (pg/ml)**	2617 (1699-4455)	2167 (1644-4053)	P=0.66
**After 19 weeks (pg/ml)**	1523 (1331-2205)	1506 (1237-1722)	P=0.85
**Wilcoxon test Baseline vs. After 19 weeks**	P=0.001	P=0.02	

Supplementary Table 3- distance traveled in EPM and OF

	**Sedentary** Median (IQR)	**Running** Median (IQR)	**Mann Whitney U-test Sedentary vs. Running**
**Distance moved Elevated plus maze**	1048 (741-1357)	1278 (964-1616)	P=0.23
**Distance moved Open field**	4569 (3115-5561)	4017 (3746-5568)	P=0.93

Supplementary Table 4- Sucrose Preference

	**Sedentary** Median (IQR)	**Running** Median (IQR)	**Mann Whitney U-test** P-value
**Sucrose Preference (%)**	79.6 (76.4-86.1)	79.7 (73.2-88.4)	1.0

Supplementary Table 5- Aβ levels (ELISA)

	**Different Aβ species**	**Sedentary** concentration (ng Aβ /mg protein) Median (IQR)	**Running** concentration (ng Aβ /mg protein) Median (IQR)	**Mann Whitney U-test** p-values prior to Bonferroni correction
**Insoluble fraction in hippocampus**	Aβ-38	164.5 (131.5-322.4)	255.3 (147.8-303.4)	0.57
	Aβ-40	1048 (712.4-1286)	1194 (989.7-1526)	0.35
	Aβ-42	8007 (5909-10173)	9612 (4285-11546)	0.78
**CSF** (n=7+7)	Aβ-38	0.59 (0.54-0.88)	0.44 (0.19-0.54)	0.21
	Aβ-40	3.87 (2.17-4.92)	2.28 (0.76-3.23)	0.32
	Aβ-42	1.81 (1.18-2.46)	1.25 (0.35-1.68)	0.32

Supplementary Table 6- Iba1 and gal-3 in hippocampus (immunohistochemistry)

	**Sedentary** Median (IQR) Fold to actin %	**Running** Median (IQR) Fold to actin %	**Mann Whitney U-test** P-value
**Galectin-3** (n=6+6)	116 (84-125)	114 (102-127)	0.94
**NLRP3** (n=3+4)	22 (18-33)	17 (16-20)	0.63

Supplementary Table 7- Cytokine levels (ELISA)

	**Cytokine**	**Sedentary** Mean concentration (pg/ml) ± SD	**Running** Mean concentration (pg/ml) ± SD	**T-test** P-values prior to Bonferroni correction
**Hippocampus**	IL-1 β	1.29 ± 0.7	1.34 ± 0.8	0.85
	IL-2	0.022 ± 0.01	0.029± 0.02	0.26
	IL-4	0.045 ± 0.02	0.052 ± 0.04	0.58
	IL-5	0.008 ± 0.003	0.010 ± 0.005	0.26
	IL-6	0.45 ± 0.3	0.68 ± 0.7	0.27
	IL-10	0.15 ± 0.09	0.15 ± 0.07	0.99
	IL-12p70	1.57 ± 0.8	1.78 ± 0.9	0.52
	IFNγ	Below detection	Below detection	
	TNFα	0.067 ± 0.02	0.082 ± 0.06	0.40
	KC/GRO	2.75 ± 1.9	2.94 ± 2.6	0.82
**Serum**	IL-1 β	0.27 ± 0.2	0.20 ± 0.2	0.37
	IL-2	0.12 ± 0.1	0.13 ± 0.1	0.84
	IL-4	Below detection	Below detection	
	IL-5	1.48 ± 1.3	1.68 ± 1.0	0.64
	IL-6	3.25 ± 2.6	3.59 ± 4.2	0.80
	IL-10	7.14 ± 3.9	6.14 ± 3.6	0.48
	IL-12p70	Below detection	Below detection	
	IFNγ	0.33 ± 0.1	0.20 ± 0.1	0.06
	TNFα	3.66 ± 1.0	4.17 ± 1.8	0.38
	KC/GRO	34.14 ± 29.8	23.52 ± 10.2	0.22

Supplementary Figure S1- Distance in running wheels

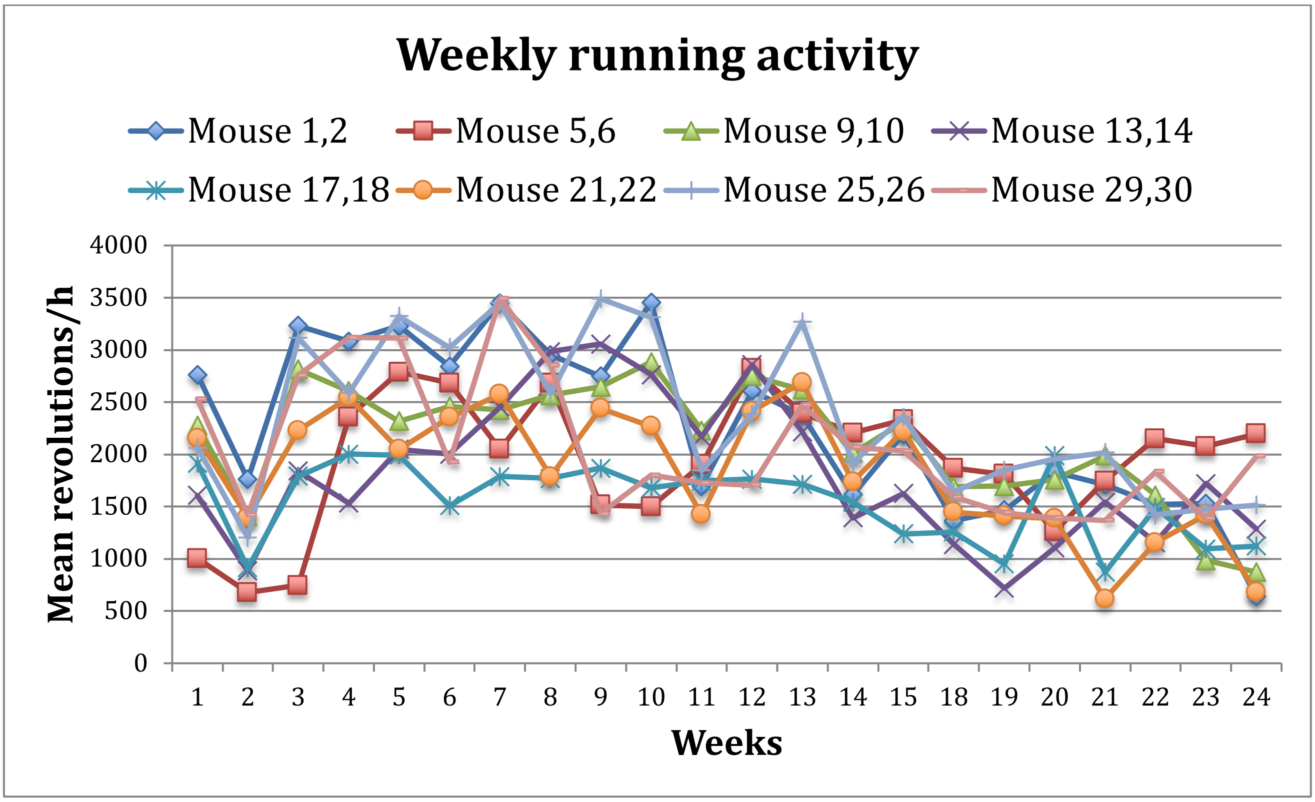

Figure S1. Running activity displayed as mean revolution per hour for each week and each couple sharing a running wheel in their home cage. Each revolution corresponds to a running distance of around 42 cm. ANOVA repeated measurements showed a change in the amount of running over time ($p<0.001$) and a paired T-test comparing the running during the first week with that of the last week of intervention revealed a trend towards decreased running over time (means±SD were 2039±549 revolutions/h during the first week compared to 1288±578 revolutions/h during the last week, paired T-test, $p=0.07$).

Supplementary Methods

Sucrose preference test

To assess anhedonic behavior, a Sucrose preference test was performed during the night before sacrifice. Mice were introduced to a sucrose solution in their home cages one night before the test. A bottle containing 2% sucrose solution was put in the place where the regular bottle with tap water used to be during the night. The regular bottle with tap water was placed in the other corner of the cage, allowing the mice to choose. The day before the test, mice were deprived from drinking five hours prior to the test. Later, mice were individually caged with access to nesting material, food pellet, as well as two bottles, one tap water and one sucrose solution as described

before [1]. Bottles were weighed before and after the test and the volume consumed was calculated. A sucrose preference index was calculated using the following formula: *Sucrose preference index=weight of consumed sucrose/total weight consumed of both solutions*

Multiplex ELISA

Cytokine ELISA

The concentrations of different cytokines in serum as well as in the pooled first and second fraction of homogenized hippocampus (25 µl/sample) were measured with the MSD Mouse Proinflammatory V-Plex Plus Kit (IFNγ, IL-1β, IL-2, IL-4, IL-5, IL-6, IL-10, IL-12p70, CXCL1, TNFα; K15012C, Mesoscale) using a QuickPlex SQ120 (Mesoscale Discovery, Rockville, USA) Plate Reader according to the manufacturer's instructions. The recorded data was analyzed using MSD Discovery Workbench software. For the brain homogenate samples, the cytokine concentrations were normalized to the total protein concentrations measured in the BCA or Bradford assay.

Aβ ELISA

The concentration of different Aβ species in the insoluble fraction of homogenized hippocampus as well as in the CSF were measured with the MSD MULTI-SPOT Human (4G8) Aβ Triplex Assay (Aβ38, Aβ40 and Aβ42; K15199G-1, Mesoscale) using QuickPlex SQ120 (Mesoscale Discovery, Rockville, USA) Plate Reader according to the manufacturer's instructions. The recorded data was analyzed using MSD Discovery Workbench software. For the brain homogenate samples, Aβ concentrations were normalized to total protein concentrations measured in the BCA or Bradford assay.

Fecal corticosterone levels

Fecal samples were collected from the Open field arena after conducting the Open field test in order to measure the stress levels of the mice. The feces were stored at -80°C until use. Corticosterone was then extracted and analyzed with a corticosterone ELISA kit (Enzo Life Sciences) described by Touma et al.[2] except that feces was homogenized in 1 ml of 80% Methanol per 100 mg sample, as we have done before[3].

References

1 Bay-Richter, C. *et al.* Behavioural and neurobiological consequences of macrophage migration inhibitory factor gene deletion in mice. *J Neuroinflammation* **12**, 163, doi:10.1186/s12974-015-0387-4 (2015).

2 Touma, C., Sachser, N., Mostl, E. & Palme, R. Effects of sex and time of day on metabolism and excretion of corticosterone in urine and feces of mice. *Gen Comp Endocrinol* **130**, 267-278, doi:S0016648002006202 [pii] (2003).

3 Svensson, M. *et al.* Forced treadmill exercise can induce stress and increase neuronal damage in a mouse model of global cerebral ischemia. *Neurobiol Stress* **5**, 8-18, doi:10.1016/j.ynstr.2016.09.002 (2016).
